# Steroid hormone regulation of innate immunity in *Drosophila melanogaster*

**DOI:** 10.1371/journal.pgen.1010782

**Published:** 2023-06-15

**Authors:** Scott A. Keith

**Affiliations:** 1 Department of Entomology, Cornell University, Ithaca, New York, United States of America; 2 Cornell Institute of Host-Microbe Interactions and Disease, Cornell University, Ithaca, New York, United States of America; Universidad de Valparaiso, CHILE

## Abstract

Endocrine signaling networks control diverse biological processes and life history traits across metazoans. In both invertebrate and vertebrate taxa, steroid hormones regulate immune system function in response to intrinsic and environmental stimuli, such as microbial infection. The mechanisms of this endocrine-immune regulation are complex and constitute an ongoing research endeavor facilitated by genetically tractable animal models. The 20-hydroxyecdysone (20E) is the major steroid hormone in arthropods, primarily studied for its essential role in mediating developmental transitions and metamorphosis; 20E also modulates innate immunity in a variety of insect taxa. This review provides an overview of our current understanding of 20E-mediated innate immune responses. The prevalence of correlations between 20E-driven developmental transitions and innate immune activation are summarized across a range of holometabolous insects. Subsequent discussion focuses on studies conducted using the extensive genetic resources available in *Drosophila* that have begun to reveal the mechanisms underlying 20E regulation of immunity in the contexts of both development and bacterial infection. Lastly, I propose directions for future research into 20E regulation of immunity that will advance our knowledge of how interactive endocrine networks coordinate animals’ physiological responses to environmental microbes.

## Introduction

Animals continuously interact with diverse populations of environmental microorganisms that profoundly impact their biology. Pathogenic microbes, in particular, can elicit rapid and dramatic changes to overall animal physiology in support of a resource-costly immune response. Endocrine signaling networks constitute one of the key mechanisms animals have evolved to rapidly modulate biological functions in response to intrinsic and extrinsic stimuli. Across metazoans, major physiological and life history traits are subject to endocrine regulation. These include growth, development, metabolism, reproduction, aging, stress responses, and immunity [1–5]. Notably, these traits are also strongly influenced by interactions with microbes [6,7]. Systemically circulating hormones can bind to receptors that are expressed in multiple distant target tissues and may regulate diverse cellular and physiological processes simultaneously. This concept of “hormonal pleiotropy” [2,8] posits hormones as principal factors that control the balance among competing biological processes, thereby enabling animals to quickly and dynamically coordinate their physiology in response to environmental conditions, including the microbial environment. Endocrine regulation of an integrated physiological response to environmental microbes therefore has profound implications for infectious disease dynamics and opens a role for microbes to shape the adaptive evolution of animal physiology [8–11].

The immune system comprises the main biological processes through which animal hosts negotiate their relationships with beneficial, commensal, and pathogenic microbes. Steroid hormone regulation of innate and adaptive immune responses is observed across taxa from invertebrates to mammals. In vertebrates, glucocorticoids, androgens, and progesterone have been shown to both positively and negatively regulate immune responses, depending on their titers and physiological conditions [12,13]. These steroids are frequently employed pharmaceutically for their anti-inflammatory properties in humans [14,15]. By contrast, estrogens primarily have immune potentiating effects, stimulating T and B cell development, activation, and cytokine production [16]. Similar endocrine-immunity regulatory relationships also occur in laboratory model animals, including mice [15], zebrafish [17], and fruit flies [1], underscoring their prevalence and facilitating experimental investigation of the underlying molecular mechanisms.

Insects comprise 80% of the estimated species biodiversity on earth [18,19], with pivotal roles as pollinators, agricultural pests, and infectious disease vectors; as such, their impacts on global human health are vast and far-reaching. Unlike vertebrates, insects possess only 1 major class of steroid hormone, the ecdysteroids, predominated by the most biologically active hormone 20-hydroxyecdysone (20E) [20]. Similarly to vertebrate steroids, 20E regulates many physiological and organismal insect traits, including juvenile-to-adult developmental transitions [3,21,22], reproductive maturation [23], energy metabolism [24], behavior [25], stress responses [26], and longevity [26,27].

20E also regulates multiple aspects of insect immunity [1]. Though long-recognized, historically this regulation has received considerably less research attention than the developmental functions of 20E. Despite potentially major implications for health-improving measures like pest and disease vector control, our understanding of 20E-mediated insect immune responses lags behind that of mammalian steroid–immunity interactions. Work over the past 20 years, however, has begun to define the mechanisms through which 20E modulates immunity, the prevalence of this regulation across organ systems and life stages, and the ultimate physiological consequences for the insect host. Here, I review significant recent studies pertaining to 20E regulation of insect innate immunity, largely within the conceptual framework of anticipatory and infection-activated defenses against bacterial pathogens. I primarily focus on work conducted using the model insect *Drosophila melanogaster* (hereafter *Drosophila*), where genetic amenability has yielded mechanistically detailed discoveries.

### Correlation between 20E signaling and immune system activation in insects

The best-studied role of 20E is driving major developmental transitions during the insect juvenile growth period, including key stages of embryogenesis [28,29], the molts between larval instars [3,21], and, in holometabolous insects, the onset of puparium formation and metamorphosis [20,30,31]. Peaks in 20E production and circulating titers initiate the irreversible cellular and morphological changes that constitute progression through the insect life stages [20]. In addition to these well-studied developmental effects, specific 20E peaks are also accompanied by activation of immune processes in a variety of insect taxa as detailed below.

Insects lack the antibody-mediated, acquired immune systems of vertebrates, but possess widely conserved innate immune mechanisms comprising the 2 distinct but functionally interconnected branches of humoral and cellular immunity. Humoral immunity involves the action of 2 minimally overlapping, microbe-responsive signaling cascades, the Toll and IMD pathways [32]. While Toll signaling is mainly activated by gram-positive bacteria and fungi, IMD responds to meso-diaminopimelic acid (DAP)-type peptidoglycan (PGN) derived from gram-negative (and some gram-positive) bacteria [33,34]. DAP-type PGN binds to and activates transmembrane peptidoglycan recognition protein (PGRP) receptors, including the major IMD-activating receptor PGRP-LC [35]. PGN-bound PGRPs initiate an intracellular signaling cascade that ultimately leads to activation and nuclear translocation of the NF-κB-like transcription factors Relish (for the IMD pathway) and Dorsal and Dorsal-related immunity factor (Dif; for the Toll pathway), which transcriptionally up-regulate expression of antimicrobial peptides (AMPs) [32,34]. Systemic bacterial infection with gram-negative pathogens leads to rapid, massive expression and secretion of AMPs, in an effort to constrain the infection through their bactericidal actions [36,37]. In parallel to this humoral response, cellular immunity is mediated by a heterogeneous population of hemocytes that contribute to immune defenses via phagocytosis of microbial cells, encapsulation of larger foreign bodies like parasite eggs, and promotion of wound healing [38,39]. Hallmarks of the activation of both cellular and humoral systems are associated with peaks in 20E abundance at critical stages in development.

**Fig 1:**
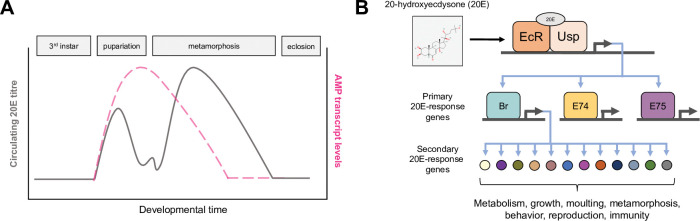
20E signaling activity during *Drosophila* development coincides with up-regulation of immune genes. **(A)** Schematic illustrating the temporal dynamics of 20E titer and immune gene induction during insect pupariation. Distinct peaks in hemolymph 20E levels drive developmental transitions including the onset of metamorphosis in *Drosophila* and other holometabolous insects [20,30,31]. A dramatic, microbe-independent up-regulation of immune gene transcripts, including AMPs, also occurs at pupariation [40,45]. (B) 20E activates a hierarchical, temporally controlled gene regulatory network. Circulating 20E binds to and activates the heterodimeric NHR complex consisting of EcR and Usp. 20E-EcR/Usp directly activates transcription of “early” response genes encoding additional NHRs and transcription factors, including *Broad* (*Br*), *Eip74EF* (*E74*), and *Eip75B* (*E75*). These NHRs up-regulate expression of “late” response genes, which code for proteins that actuate a variety of cellular and physiological processes [20,22,81]. 20E chemical structure depiction from PubChem [151]. AMP, antimicrobial peptide; EcR, ecdysone receptor; NHR, nuclear hormone receptor; Usp, ultraspiracle; 20E, 20-hydroxyecdysone.

In *Drosophila*, studies profiling the genome-wide expression changes that occur at 20E-dependent developmental transitions have consistently documented increased expression of genes coding for humoral immune pathway components [40–43]. This correlation is prominent during pupariation and the initiation of metamorphosis, the stage at which circulating 20E titers are higher than any other point of the insect lifespan [3,30,44]. Transcript levels of genes encoding the PGRPs that stimulate each the IMD and Toll pathways, core signal transduction factors in those pathways, and the transcription factors *Relish*, *Dorsal*, and *Dif* are elevated in early pupae relative to the preceding larval stages [41,45]. Most strikingly, however, both Toll- and IMD-regulated AMP transcripts spike dramatically during pupariation relative to their levels at preceding and subsequent life stages (Fig 1A) [40,42,45–50].

In addition to the larval-to-pupal transition, a parallel between 20E signaling activity and humoral immune function has also been identified in *Drosophila* embryos. During embryogenesis, a 20E signaling peak beginning at stage 12 drives germ band retraction [28]. Tan and colleagues [51] found that stage 11 embryos, in which 20E levels are low, failed to induce *Drosocin* (*Dro*) and *Cecropin A1* (*CecA1*) expression, constrain bacterial loads, and survive when injected with *Ecc15*. By contrast, stage 15 embryos mounted a robust AMP response and survived infection at higher proportions [51]. Thus, 20E levels correspond to humoral immune response capacity at multiple stages of *Drosophila* development.

Similar correlations between developmental 20E pulses and immune induction have been observed in other insect taxa. Transcripts of PGRPs and AMPs increase in fat body tissues of wandering *versus* feeding larvae of the cotton bollworm *Helicoverpa armigera* [52]. *Hemolin*, which encodes a Lepidopteran-specific, infection-stimulated immunoglobulin peptide, is also transcriptionally elevated in wandering *H*. *armigera* and *Manduca sexta* larvae [52–54]. Immune gene transcript abundance also increases alongside developmental 20E peaks in nymphs of the hemimetabolous locust *Locusta migratoria* [55]. Johnston and colleagues [56] recently documented an up-regulation of a limited number of immune genes at metamorphosis in the gut of the wax moth *Galleria mellonella*. In addition to humoral immune induction, major changes to cellular immune processes occur during developmental 20E peaks. At puparium formation in *Drosophila*, phagocytic hemocytes increase in number and size, and previously sessile, integument-attached cell populations disperse widely throughout the animal’s body [57–59]. Similar changes in hemocyte properties at pupariation have been reported for *Calliphora vicina* larvae [60].

Functional modulation of both humoral and cellular immunity at key 20E-regulated developmental time points thus appears to be broadly conserved across insects. The prevalence of immune induction at metamorphosis has informed the hypothesis that 20E-induced AMP production evolved to prevent infection at a life stage that renders insects particularly vulnerable to microbial pathogens. More specifically, a prophylactic model has been proposed wherein 20E signaling drives both the extreme morphological changes that sensitize the insect to pathogens and robust AMP induction that limits bacterial burden and protects against infection [1,40,56,61–63]. Larvae of holometabolous insects are immersed in and continually feed on food substrates containing diverse microbial populations [64]. Food-associated microbes are therefore carried within the larval alimentary tract into the metamorphic process [40,65], during which the gut undergoes dramatic remodeling events, including delamination [66–68], and 16S rRNA gene profiling studies have revealed a marked decrease in bacterial abundance within the pupal case [40,65], consistent with the microbicidal activity of strong immune activation. Nunes and colleagues [40] recently showed that AMP up-regulation in early pupal stage *Drosophila* also occurs in flies reared under germ-free (microbiologically sterile) conditions, suggesting that 20E-driven immune activation at pupariation may represent an intrinsic regulatory mechanism independent of microbial stimulus. Importantly, however, other studies have shown that 20E suppresses developmental induction of immune genes in the silkworm *Bombyx mori* [69] and the blowfly *C. vicina* [70]. Thus, while innate immune activation at metamorphosis appears widespread across holometabolous insects, this activation may not be driven by 20E in all species.

Overall, the close temporal correlation between high 20E titers and immune induction at the wandering and metamorphic insect stages led to the prediction that 20E signaling might directly and positively regulate innate immune pathways. Below, I discuss studies directly testing these hypotheses through functional experiments, which have revealed evidence indicating direct regulation of immune function by 20E.

### 20E signaling regulates immune activation during development and infection

Over 5 decades of research on 20E function in *Drosophila* has yielded important, generalized discoveries about how hormones regulate gene expression, cell biology, and organismal physiology [3,20–22]. Like mammalian steroid hormones, 20E modulates genome-wide expression changes in target tissues by binding to a nuclear hormone receptor (NHR) complex. Cellular uptake of circulating 20E was recently shown to require a membrane transporter, the Ecdysone Importer (also known as Oatp74D), originally discovered in *Drosophila* and subsequently found to facilitate 20E signaling in other insects [71–74]. Intracellular 20E binds to and activates a heterodimeric NHR comprising the proteins ecdysone receptor (EcR) and ultraspiracle (Usp) [3,20–22,75]. The EcR/Usp complex binds to ecdysone response element (EcRE) sequences in the promoters of target genes, thereby either repressing or activating the transcription of those genes [76–79]. Many 20E-EcR/Usp target genes encode additional NHRs and transcription factors, including the canonical “early” 20E response targets *Eip74EF*, *Eip75B*, *E93*, *Hr3*, and *Broad* [80–83] and several more genes [77,78,84]. These NHRs in turn regulate an extensive set of downstream pathways and processes by modulating the expression of their own target gene regulons. Thus, 20E activates a hierarchical transcriptional program consisting of primary and secondary responses that are directly and indirectly initiated by EcR/Usp (Fig 1B) [20,22,81].

In the larval and pupal stages, 20E activation of EcR/Usp regulates a variety of distinct processes depending on cell and tissue context, including organ growth, remodeling, and apoptosis [3,22,67,68]. Similarly, experiments primarily utilizing genetic approaches in *Drosophila* have demonstrated a functional requirement for 20E signaling to fully activate immune responses. These functional connections have been observed in multiple cell and tissue types both during development [40,51,58,85] and in response to infection [41,51,58,86–89].

#### Fat body

The insect fat body is a highly versatile organ that is functionally comparable to the mammalian liver and adipose tissue, with roles in energy metabolism, xenobiotic detoxification, and egg provisioning to support reproduction [90]. The fat body is also the primary immune-responsive tissue in reaction to systemic pathogen challenge, producing AMPs at extremely high levels and secreting them into the hemolymph [32,90–93]. 20E signaling in the fat body appears to be important for immune activation during infection in adult flies. Rus and colleagues [41] showed that EcR and a suite of 20E early genes, including *Br*, are each independently required in the adult fat body for pathogen-induced up-regulation of *PGRP-LC*, *CecA1*, and *Dpt*. Further, RNAi-mediated depletion of EcR or any of these primary targets in the fat body led to elevated bacterial burdens and dramatically increased lethality caused by the typically minimally virulent *Drosophila* pathogen *Pectinobacterium* (*Erwinia*) *carotovora* (*Ecc15*). A notable exception was the early gene *Eip75B*, a fly NHR with sequence homology to mammalian Rev-ErbA [94] that mediates responses to the PPARγ agonist Pioglitazone [95,96]. Fat body RNAi knockdown of *Eip75B* enhanced AMP transcript levels, accelerated pathogen clearance, and increased survival [41]. This finding raises the intriguing possibility that 20E-EcR can both potentiate and suppress immune responses in the fat body through pleiotropic regulation of its suite of downstream transcription factors.

Beyond systemic infection in adult flies, Nunes and colleagues [40] provide strong functional evidence that 20E signaling to the larval fat body contributes to AMP induction at *Drosophila* pupariation. The authors showed that genetically disrupting EcR function specifically in the fat body fully abrogates the whole-animal transcriptional increase in *Drs* at pupariation, indicating a requirement for fat body 20E signaling to up-regulate this AMP. Further epistasis experiments revealed that the early 20E response gene and direct EcR/Usp target *Broad* (*Br*) is required downstream of EcR to drive metamorphic *Drs* induction. Interestingly, the authors also found that loss of fat body EcR function yielded pupae carrying higher loads of *Acetobacter* and *Lactobacillus*, the predominant genera of the *Drosophila* gut microbiota [64,65]. Collectively, this study’s compelling results support the model wherein developmental, 20E-dependent immune induction may have evolved to constrain bacterial burden during metamorphosis.

Together, these reports indicate that 20E signaling to the fat body, the principal organ of insect innate immunity, regulates immune activation both developmentally and during infection, via complex but as yet poorly understood mechanisms with important physiological consequences for the animal.

#### Hemocytes

Hemocytes are present for the duration of the fly’s life cycle, from the embryonic through adult stages, and play critical roles in mitigating bacterial and parasitic infection, wound repair, and clearing of apoptotic cell debris generated by developmental processes [39,97–101]. In larvae, 20E signaling regulates the production, differentiation, motility, and phagocytic activity of hemocytes in developmental and infection contexts. In pre-metamorphic larvae, the circulating hemocyte population is maintained by hematopoietic divisions of progenitor cells in the larval lymph gland [39,100]. Ramesh and colleagues [85] recently showed that 20E signaling to the lymph gland maintains the appropriate number of hematopoietic precursor cells by regulating the expression levels of *Relish*. Loss of EcR function in the hematopoietic niche cells of the lymph gland leads to decreased levels of *Relish* transcript and protein in these cells, resulting in over-proliferation and precocious differentiation of hemocytes, with associated decrease in the maintained pool of progenitors [85].

At the onset of metamorphosis or in response to infection, larval hemocytes increase in numbers, motility, and phagocytic capability [39,57–59,100]. Hemocytes extracted from pre-metamorphic third instar larvae and treated with exogenous 20E can be induced to undergo changes paralleling those observed during development, strongly resembling hemocytes isolated from early pupae [59]. Notably, 20E treatment also induces morphological changes and increases the phagocytic activity of l(2)mbn cells [102], a cell culture line derived from the larval hemocytes of a tumorous *Drosophila* mutant [103]. Furthermore, blocking 20E signaling specifically in the hemocytes disrupts their proliferation, motility, and phagocytic activity in response to exogenous 20E treatment [59] as well as in vivo during pupariation [58]. Regan and colleagues [58] showed that this cell autonomous, EcR-dependent activation is required for hemocytes to phagocytose bacteria and prevent mortality during systemic infection in pupae. These findings collectively suggest that, in addition to humoral AMP production, 20E-driven hemocyte activation also contributes to prophylactic immune induction at metamorphosis, protecting the insect from bacterial infection at this sensitive life stage.

#### Gut

The *Drosophila* intestine is a functionally compartmentalized, cellularly heterogeneous epithelium that continuously interacts with ingested, luminal microbes [64,66]. Both the commensal microbiota and enteric pathogens induce a localized, humoral immune response in the gut, with effects on intestinal stem cell division, metabolism, development, reproduction, and longevity [104–109]. In fly larvae, which dwell in and continually feed on the microbe-rich food substrate, the gut microbiota are a critical determinant of growth rate and growth capacity. Transcript levels of the 20E biosynthetic enzymes *shroud* and *shade* [110] and the early gene *Eip74B* [111] have been reported to decrease in germ-free larvae, suggesting interactions with the gut microbiota can affect expression of certain 20E-related genes during development. At pupariation, Nunes and colleagues [40] found that 20E-EcR function in the larval gut (similarly to the fat body) up-regulates multiple AMPs, thereby protecting against excess microbiota loads in late pupae. The 20E spike that drives pupation therefore additionally activates a localized immune response in the late larval/early pupal gut that constrains luminal bacterial populations, insulating the insect from perhaps the most likely route by which commensal gut microbes could infiltrate metamorphosing insects and become conditionally pathogenic.

Work by multiple labs published within the past 3 years has revealed that 20E also modulates intestinal physiology in adult female flies, a previously unrecognized function of this hormone [86,87,95,112]. Jugder and colleagues [87] found that 20E signals to a specific population of gut cells to activate the IMD pathway in response to microbiota-derived small molecules, which in turn suppresses aberrant intestinal lipid accumulation. A previous report by the same group had shown that acetate produced by the *Drosophila* microbiota promotes PGRP-LC-dependent IMD signaling in enteroendocrine cells (EECs) of the gut, which in turn prevents lipid droplet accumulation in enterocytes via activation of the insulin/insulin-like growth factor (IIS) signaling pathway [107]. Building on this study, Jugder and colleagues [87] present compelling genetic evidence that the mechanism of acetate-induced IMD activation in EECs (and consequent downstream prevention of excess intestinal lipid accumulation) involves 20E-EcR-dependent transcriptional up-regulation of *PGRP-LC*. This transcriptional regulation may occur via an interaction (direct or indirect) between the EcR/Usp complex and the Tip60 chromatin remodeling complex, which uses acetate-derived acetyl-CoA for histone acetylation, and could plausibly facilitate chromatin accessibility of the PGRP promoter, enabling 20E-EcR/Usp-controlled expression induction [87]. Whether this proposed mechanism might contribute to other microbe-responsive gut functions warrants future investigation. Intriguingly, Ahmed and colleagues [86] recently showed that intestinal stem cell proliferation activated by enteric infection with the highly virulent, gram-negative pathogen *Pseudomonas entomophila* requires Usp and Eip75B, but not EcR, further suggesting complex, novel mechanisms whereby 20E controls microbe-induced physiological changes in the adult intestine.

#### Malpighian tubules

Malpighian tubules are branched tubules that constitute the insect renal system, extending from the posterior gut. In addition to and intertwined with their primary excretory functions, malpighian tubules are immune-responsive organs with critical roles in maintaining physiological homeostasis subsequent to pathogen challenge [88,89,113,114]. Verma and Tapadia [89] showed that tissue-autonomous transcriptional induction of AMPs in both larval and adult malpighian tubules requires 20E signaling. Specifically, they found that 20E-EcR/Usp signaling in malpighian tubules leads to Br-dependent up-regulation of Relish transcript and protein levels, facilitating robust AMP induction after ex vivo malpighian tubules are exposed to PGN. Further, RNAi depletion of Br in malpighian tubules increased the susceptibility of adult flies to oral infection with *Escherichia coli* [89]. Similar in vivo results were reported by Zheng and colleagues [88]. These authors found that adult flies preexposed to mild desiccation stress and subsequently allowed to recover exhibited increased resistance to systemic bacterial infection. Desiccation treatment stimulated 20E production, and EcR and PGRP-LC were jointly required in the malpighian tubules for the observed improvement in survival of infection after desiccation [88]. Together, these studies indicate that 20E-dependent activation of IMD in malpighian tubules is required for full resistance to both enteric and systemic infections.

#### Tracheae

The tracheal system in insects comprises an elaborately branched tubular network that serves as the primary site of respiratory gas exchange between the tissues and the external environment [115]. Similar to other epithelia, tracheal cells express AMPs both constitutively and in response to microbial challenge [51,93,116]. As previously mentioned, Tan and colleagues [51] found that *Drosophila* embryos expressed AMPs predominantly in tracheal tissue in response to infection, but were only competent to do so in later stages of embryogenesis, subsequent to a developmental 20E pulse. However, the authors found that treating embryos with exogenous 20E at an earlier developmental stage rendered them capable of mounting this tracheal immune response, and showed that EcR function was specifically required in tracheae of late embryos to induce AMPs, constrain bacterial loads, and enable survival following bacterial infection. This study demonstrates that stage-dependent 20E pulses that drive key morphological events during embryogenesis can also prime the embryonic tracheal tissue to respond to and resist pathogens.

#### Summary

Altogether, the work discussed in this section shows that circulating 20E acts through its receptor and downstream signaling targets to activate both the humoral and cellular arms of the *Drosophila* innate immune response in multiple organ systems during development and infection. Commonalities in regulatory modes across multiple tissue types have emerged, including Br-dependent up-regulation of both upstream signaling pathway factors and AMP effector genes [40,41,89]. However, the precise molecular mechanisms by which 20E exerts these effects are less clear from the extant in vivo studies. As detailed in the next section, work conducted in cell culture has been powerful for revealing transcriptional regulation of immune-related genes by the 20E-EcR signaling axis.

### Molecular mechanisms of immune gene regulation by 20E signaling

Experiments in *Drosophila* cell culture lines offer a highly tractable system to test molecular genetic hypotheses [117]. A long-standing observation that exogenous 20E treatment strongly potentiates immune responses dates to some of the earliest work establishing immortalized *Drosophila* cell lines as tools for immunity research [103,118]. Specifically, cells preincubated with purified 20E show robust AMP expression following application of microbe-derived stimuli including purified bacterial cell wall components like peptidoglycan (PGN) or lipopolysaccharide (LPS), while cells not pre-treated with 20E exhibit a weak response [41,46,102,119–121]. This requirement for 20E to mount a full immune response has been observed in l(2)mbn cells [46,102] and in the widely used Schneider 2 (S2) cell line, which is derived from embryonic macrophages [41,118,120,121].

**Fig 2:**
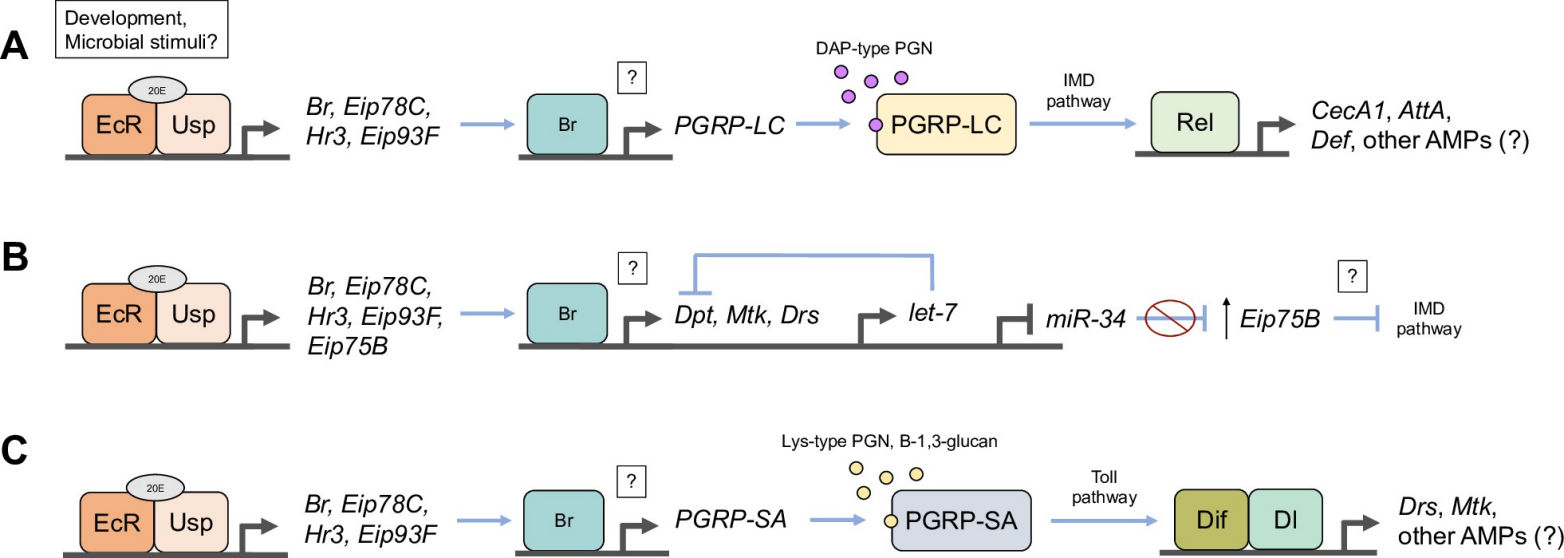
Proposed transcriptional regulatory mechanisms through which 20E signaling can promote immune activation and AMP expression. The 20E-EcR/Usp complex induces expression of early genes encoding additional transcription factors including Br, Eip78C, Hr3, Eip93F, and others, each of which is required for 20E-dependent immune gene induction [40,41,89]. **(A)** 20E-activated transcription factors such as Br and other NHRs may directly regulate and increase the expression of the PGRP-LC receptor, which facilitates IMD signaling in response to DAP-type peptidoglycan (PGN) derived from gram-negative bacteria. IMD signaling downstream of up-regulated PGRP-LC results in Relish (Rel)-dependent transcriptional induction of IMD-regulated AMPs [41,87,89]. **(B)** 20E-activated NHRs may bind to *cis* regulatory sequences in the promoters of particular AMPs and thereby directly induce their up-regulation in response to as yet unknown intrinsic (developmental) or external (microbial) signals [40,41,46]. Direct regulation of immune genes including *PGRP-LC* and various AMPs by 20E-regulated transcription factors warrants further investigation. Detailed mechanistic studies have shown that Br regulates expression of the miRNAs *let-7* and *miR-34* [121,124]. Br promotes expression of *let-7*, which directly targets *Dpt* transcripts [124]. *miR-34* activates immunity in part by targeting transcripts of the 20E-induced negative IMD regulator *Eip75B* [121]. 20E signaling via Br suppresses *miR-34* expression, derepressing *Eip75B* which diminishes IMD activity through unclear mechanisms that might involve *PGRP-LC* suppression [41,121]. **(C)** 20E could potentiate induction of Toll-responsive AMPs by driving expression of genes coding for Toll pathway components, such as the activating receptor PGRP-SA [55] or fungal-responsive proteins like GNBP1 and GNBP3. Analogous to the PGRP-LC-dependent mechanism **(A)**, this could facilitate AMP up-regulation via Dorsal-related immunity factor (Dif)-Dorsal (Dl) activation downstream of canonical Toll signaling [40]. This putative mechanism and the cellular/physiological contexts in which it might occur have not been thoroughly investigated in *Drosophila*. AMP, antimicrobial peptide; EcR, ecdysone receptor; NHR, nuclear hormone receptor; PGRP, peptidoglycan recognition protein; Usp, ultraspiracle; 20E, 20-hydroxyecdysone.

Through a series of elegant experiments investigating the mechanistic basis of 20E-potentiated AMP expression, Rus and colleagues [41] determined that 20E signaling through EcR and multiple early gene transcription factors drives *PGRP-LC* expression. This 20E-induced increase in basal *PGRP-LC* levels effectively primes the IMD pathway to rapidly respond upon encountering microbial stimuli like PGN, thereby enhancing downstream AMP production. A crucial finding supporting this model was that PGRP-LC overexpression in S2 cells obviated the 20E requirement for PGN-stimulated *CecA1*, *AttA*, and *Def* induction. However, PGRP-LC overexpressing cells still fully required 20E pretreatment for PGN-induced up-regulation of 3 different AMPs: *Dpt*, *Mtk*, and *Drs*. While *Drs* is predominantly Toll-regulated [36,122,123], the failure to up-regulate *Dpt* and *Mtk* is particularly intriguing because the authors also showed that PGRP-LC overexpression in the absence of 20E is still sufficient to drive the IMD signaling cascade, including Relish cleavage, phosphorylation, and nuclear localization. Together, these results suggest 20E signaling can promote expression of distinct subsets of AMPs via multiple regulatory mechanisms, for which there are varying amounts of additional evidence (Fig 2).

One mechanism supported by Rus and colleagues’ [41] findings involves transcriptional up-regulation of *PGRP-LC* via 20E-EcR/Usp-induced early genes, which in turn facilitates enhanced signaling through the IMD pathway, leading to Relish-driven up-regulation of AMPs including *CecA1*, *AttA*, and *Def* (Fig 2A). Another possible mechanism is direct transcriptional regulation of AMPs by 20E-regulated early genes encoding downstream nuclear receptors and transcription factors (Fig 2B). Consistent with this mechanism, Zhang and Palli [46] identified a 26 base pair sequence within the *Dpt* promoter that was necessary for 20E potentiation of PGN-induced *Dpt* up-regulation in l(2)mbn cells. Gel shift assays revealed that this sequence could be bound by nuclear protein extracts in a 20E-dependent, PGN-responsive manner and, importantly, that this binding was not outcompeted by co-incubation with excess probe of canonical EcRE DNA. The latter result suggests this sequence may be a direct target for downstream 20E-regulated transcription factors, as opposed to EcR/Usp. Subsequent motif enrichment analyses identified an 8 nucleotide sequence within the 20E-responsive *Dpt cis* regulatory region that also occurs in the promoters of *CecA1*, *AttA*, *Mtk*, and *Drs* [46]. In addition to Zhang and Palli’s [46] work, alongside evidence that Br is required for developmental *Drs* induction, putative Br binding sites have been identified in the *Drs* regulatory region via bioinformatics analysis [40]. Collectively, these findings raise the intriguing possibility that 20E-dependent activation of certain AMPs, including *Dpt*, *Mtk*, and *Drs*, could occur via their direct transcriptional regulation by EcR/Usp-induced transcription factors like Br, and possibly others (Fig 2B). Importantly, direct transcriptional regulation of *PGRP-LC*, specific AMPs, or any other immune-related genes by either EcR/Usp or 20E-regulated transcription factors/NHRs has yet to be thoroughly documented experimentally, both in cell culture and in vivo. Given the highly suggestive observations described above, future research should prioritize testing for these regulatory interactions.

20E-dependent induction of certain AMPs could also involve transcriptional activation of upstream Toll pathway components (Fig 2C). In line with this, Nunes and colleagues [40] found that induction of *Drs* at pupariation was abrogated in flies mutant for the Toll pathway NF-ΚB *Dorsal-related immunity factor* (*Dif*), suggesting 20E-driven developmental *Drs* up-regulation may be partially Toll-dependent. Additionally, Han and colleagues [55] recently reported 20E-dependent expression of the Toll pathway PGN receptor PGRP-SA, and consequent developmental AMP induction, in the locust *Locusta migratoria*. However, the prevalence and mechanisms of 20E-driven Toll pathway activation in *Drosophila* and other insects remains largely unexplored relative to IMD signaling and warrants further investigation.

Another mechanism elucidated in cell culture experiments and subsequently observed in vivo is microRNA (miRNA) modulation of 20E-immune interactions (Fig 2B). Working with S2 cells, Garbuzov and Tatar [124] found that 20E acts via EcR and Br to induce expression of the miRNA *let-7*, which dampens *Dpt* transcript levels by directly targeting the 3′ UTR. Subsequent work by Xiong and colleagues [121] showed that another miRNA, *miR-34*, further contributes to a 20E-mediated feedback network that affects immunity. Sophisticated biochemical and genetic assays in S2 cells revealed that Br, expressed downstream of 20E-EcR/Usp, directly represses *miR-34* expression by binding to *cis* regulatory sequences proximal to its genomic locus. Additional experiments revealed that *mir-34* promotes immune activation both in cells treated with PGN and in adult flies infected with *Ecc15*. Interestingly, this immune up-regulation required *miR-34*-targeted down-regulation of the 20E early gene *Eip75B*, reminiscent of Rus and colleagues’ [41] finding that RNAi depletion of *Eip75B* enhanced the survivorship of *Ecc15*-infected flies. Collectively, these findings suggest a model wherein 20E-EcR/Usp up-regulates Br, which in turn simultaneously activates expression of immune genes [40,41,89] and represses expression of a miRNA with positive IMD regulatory functions, effectively dampening the immune response in parallel. Further, *miR-34* activates immunity, at least in part, by targeting additional 20E-induced early genes, suggesting a complex feedback mechanism. As the authors postulate, this regulatory network might enable precise temporal control of 20E-driven immune responses over the course of an infection, possibly curtailing an extended inflammatory state that could be deleterious to the animal’s health and fitness [125–127].

In summary, multiple transcriptional regulatory mechanisms for 20E-dependent immune gene activation are suggested by a range of evidence from cell culture, in vivo, and in silico studies (Fig 2). Importantly, these mechanisms are not mutually exclusive, and future research should interrogate their relative contributions to 20E-potentiated immunity under different physiological conditions.

### Outlook: towards an integrated view of endocrine-mediated physiological responses to the microbial environment

The literature reviewed here demonstrates that 20E generally activates *Drosophila* cellular and humoral immunity via multipronged transcriptional regulatory functions in a variety of organs spanning the fly life cycle. Many questions remain concerning both the mechanistic details and physiological consequences of this regulation. For example, the spatiotemporal dynamics of immune regulation by 20E have yet to be determined. These dynamics may be of particular interest during the early stages of systemic bacterial infection, when rapid immune induction is crucial [128]. Circulating 20E could plausibly contribute to the distinct expression kinetics among individual AMPs subsequent to infection [36,37,93,129] by modulating IMD pathway function across different tissues. This tissue-specific functional plasticity could occur through interactions between the EcR/Usp complex and transcriptional coactivators or corepressors, a well-documented mechanism by which 20E pleiotropically controls varied developmental processes [22,130,131] that may also apply to developmental regulation of immune processes [40]. Candidate co-regulators that could be important for 20E modulation of immunity could include the Putzig-NURF chromatin remodeling complex, which physically interacts with EcR and suppresses hemocyte proliferation [131], and the EcR co-activator Taiman, which was recently shown to drive apoptotic events in developing pupal wing tissue via activation of Toll and suppression of IMD [132].

The recent discovery of Ecdysone Importer (EcI) proteins, found across dipteran clades, suggests another potential mechanism for tissue-distinct 20E immune responses [71–74]. The *Drosophila* genome encodes 4 EcI transporters with empirically demonstrated 20E uptake function: *EcI* and *EcI-2*, *-3*, and *-4* [71,72,74]. Notably, while only *EcI* is essential for development in *D*. *melanogaster*, these 4 importers exhibit distinct expression profiles across larval stages and different organ systems [71,72]. Potential roles for any of the EcI proteins in mediating 20E-regulated immune responses have not been investigated, but future studies could test the hypothesis that differences in 20E transporter expression across cell types and physiological contexts contributes to some of the phenomenological differences discussed in this review. More broadly, continued utilization of *Drosophila* genetic tools to precisely manipulate the function of 20E signaling-related genes, including *EcI*s and EcR co-regulators, in specific tissues at varied time points post infection will enrich our understanding of the mechanisms through which 20E affects pathogen-induced immune responses across organ systems.

As discussed above, 20E regulation of innate immunity appears widespread across insect taxa [1]. Consequently, investigating this regulation in *Drosophila* will yield discoveries with potentially major implications for insect disease vectors and human health. Recent studies have shown that, as in *Drosophila*, 20E potentiates immune activity in the malaria-transmitting mosquito *Anopheles gambiae* [133–136]. Female *An*. *gambiae* injected with exogenous 20E displayed enhanced immune gene expression and decreased bacterial and *Plasmodium* pathogen burdens [134,135]. Topical application of the 20E agonists methoxyfenozide or halofenozide similarly reduced *Plasmodium* infectivity [133,136], though the latter compound may achieve this reduction independently of immune stimulation [133]. Thus, chemical amplification of endogenous mosquito 20E signaling may provide a novel approach to curtail malaria transmission [reviewed in 137]. Notably, Hun and colleagues [72] recently found that *Aedes aegypti* mosquitoes, which spread severe disease-causing viruses including Zika and dengue, possess orthologs of the *Drosophila EcI-2*, *-3*, and *-4* genes but lack an *EcI* ortholog. In line with this, only *EcI* is essential for *Drosophila* larval maturation, while *Ae*. *aegypti* development and reproduction require *EcI-2* and *EcI-4*, respectively [72]. This observation raises the possibility to interfere with 20E uptake and downstream physiological functions in specific disease-carrying insect taxa. Continued investigation of the mechanisms underlying 20E-mediated immune and antimicrobial effects will aid the development of these nascent vector control strategies.

The immune response does not occur in isolation, but is interconnected with the overall physiology of the animal. Given that 20E is a highly pleiotropic hormone, another major unresolved question is whether 20E also impacts infection outcome indirectly through its effects on other physiological processes, such as metabolism and reproduction, in addition to its role in direct regulation of immunity. The massive production of AMPs following systemic infection is extremely energetically costly [92], and 20E could support immunity by signaling to metabolic organs like the gut and fat body to mediate catabolism of energy stores that fuel AMP synthesis. Additionally, reproduction and immunity are functionally linked, with reproductive activity generally being associated with suppressed immune capabilities [2,138–142]. In adult female *Drosophila*, 20E is produced by the ovaries in response to mating [86,95] and sustains multiple steps of oogenesis [143–145]. 20E signaling within the germline could therefore have an indirect, suppressive effect on immunity by promoting energetic investment in reproduction, occurring simultaneously with and in parallel to its immune activation functions in organs like the fat body, hemocytes, gut, and malpighian tubules. In line with this, Wang and colleagues [146] recently reported that in female *Ae*. *aegypti* mosquitoes, 20E signaling activated by ingestion of a blood meal up-regulates the IMD suppressor protein Pirk-like, which both dampens immune activation in the fat body and promotes ovary development and egg laying. Additional experimental examination of 20E-mediated immune–reproduction interactions across multiple insect taxa is ripe for future study. More generally, 20E is likely to modulate immunity in complex and multifarious ways extending beyond the positive transcriptional regulation of immune genes that thus far has received the greatest research attention. These 20E-mediated effects may involve the coordinated regulation of multiple biological processes across functionally diverse organ systems to directly and indirectly alter immune function. This is in line with the general property of hormones as chemical messengers that facilitate inter-organ crosstalk and physiological integration [147]. Future investigations should attempt to untangle the ways in which 20E could regulate immunity within the overarching physiological context of the infected insect and determine how environmental variables might add an additional layer of complexity to these regulatory relationships.

Lastly, exploration of 20E-mediated host–microbe dynamics should expand beyond studying the independent functions of this hormone to consider 20E activity within the larger endocrine networks that determine animal physiology. Indeed, other major *Drosophila* hormone systems, including the insulin/insulin-like growth factor signaling (IIS) pathway and the sesquiterpenoid Juvenile Hormone (JH), also affect immunity, development, metabolism, and infection outcome [120,141,148,149]. During the larval life stages, 20E, JH, and IIS interact via complex feedback systems that can be cooperative or antagonistic depending on timing and tissue contexts to drive developmental growth. These regulatory relationships are likely also relevant to developmental immune induction and in response to microbial infection at the larval and adult life stages. For example, JH has an immune-suppressive effect in *Drosophila* [120,141,150] that contrasts with immune activation by 20E, and cell culture evidence suggests JH can antagonize 20E’s immune-stimulatory properties [120]. These counterbalancing relationships have not been examined in vivo. An imperative long-term goal of this field should be to understand how these endocrine signaling pathways interact across organ systems and biological scales, spanning the chemical to the organismal, to control immunity and other aspects of animal physiology. While this integrated view is inherently more challenging to study, genetically tractable model organisms like *Drosophila* will enable discoveries about endocrine–immune relationships which can then be investigated for evolutionary conservation in other insect taxa. Further, these studies will likely uncover mechanistic principles that can be broadly applied to the pleiotropic actions of hormones across animal–microbe interactions [2]. As such, work in this exciting and developing field will yield key insights about how the microbial environment can exert evolutionary pressures on metazoans and may lead to new therapeutic strategies to combat infectious diseases.
